# Unrestricted kinematic alignment on the sagittal plane: Posterior tibial slope and combined flexion do not have boundaries to respect in terms of short‐term clinical outcomes and safety

**DOI:** 10.1002/ksa.70136

**Published:** 2025-10-28

**Authors:** Edoardo Franceschetti, Pietro Gregori, Stefano Campi, Marco Spatuzzi, Giancarlo Giurazza, Andrea Tanzilli, Biagio Zampogna, Matteo Formica, Umile Giuseppe Longo, Rocco Papalia

**Affiliations:** ^1^ Fondazione Policlinico Universitario Campus Bio‐Medico Roma Italy; ^2^ Department of Medicine and Surgery Research Unit of Orthopaedic and Trauma Surgery Roma Italy; ^3^ Orthopedics Clinic IRCCS Ospedale Policlinico San Martino, University of Genoa Genova Italy; ^4^ Department of Surgical Science (DISC) University of Genoa Genova Italy

**Keywords:** combined flexion, posterior tibial slope, sagittal alignment, total knee arthroplasty, unrestricted kinematic alignment

## Abstract

**Purpose:**

Personalized alignment in total knee arthroplasty (TKA) is gaining traction as a surgical philosophy. True personalization, however, must encompass not only the coronal plane but also the sagittal and axial planes. This study evaluates influence of the Tibial Slope (PTS) and combined flexion (CF) on outcomes in TKA performed using unrestricted kinematic alignment (KA). The aim of the study was to examine the relationship between PTS or CF and both functional outcomes and complication rates.

**Methods:**

A retrospective review was conducted on 137 patients who underwent primary TKA using KA between November 2022 and June 2023. CF was calculated by combining PTS and DFF values. Patients were categorized into groups based on CF angle (≤7.5° vs. >7.5°) and PTS (<5, between 5 and 10, >10). Clinical outcome measures (KSS, OKS, SF12 and FJS), radiographic alignment parameters and complication rates were analyzed.

**Results:**

There were no statistically significant differences between groups regarding implant survivorship, revision rates, mechanical failure, or clinical outcome scores. CF or PTS did not correlate with postoperative coronal alignment parameters or with patient‐reported outcome measures (PROMs). Additionally, the change in clinical scores from preoperative to postoperative assessments did not differ significantly between groups, indicating comparable clinical improvement regardless of CF or PTS.

**Conclusions:**

The findings suggest that restoration of native femoral and tibial flexion within a kinematic alignment does not adversely affect outcomes or increase complication rates, providing similar clinical benefits regardless the prearthritic CF or tibial slope of the patients, supporting the short‐term safety of this “unrestricted” approach in the sagittal plane.

**Level of Evidence:**

Level IV.

**Abbreviations**
CF: combined flexion
DFF: distal femoral flexion
FJS: Forgotten Joint Score
KA: kinematic alignment
KSS: Knee Society Score
LDFA: lateral distal femoral angle
MA: mechanical alignment
mHKA: mechanical hip‐knee‐angle
MPTA: medial proximal tibial angle
OKS: Oxford Knee Score
PROMs: patient‐reported outcome measures
PTS: posterior tibial slope
ROM: range of motion
SF12: Short Form 12
TKA: total knee arthroplasty

## INTRODUCTION

While current strategies in total knee arthroplasty (TKA) predominantly emphasize coronal alignment of prosthetic components [15, 16, 18], emerging evidence suggests that a truly personalized alignment approach must incorporate all three anatomical planes. Comprehensive consideration of the axial and sagittal planes, in addition to the coronal plane, is essential to optimize biomechanical outcomes [1, 2, 17]. Sagittal alignment of the femoral component is a critical determinant of both functional outcomes and long‐term survivorship in TKA [12, 39]. Excessive femoral component extension elevates patellofemoral joint contact forces and increases the risk of anterior knee pain, whereas excessive flexion is associated with a higher likelihood of implant failure [30, 32]. A key challenge in kinematically aligned‐TKA (KA‐TKA) lies in achieving accurate sagittal alignment of the tibial component. In contrast to mechanical alignment (MA), which generally aims for a standardized distal femoral flexion (DFF) angle of 0° and a tibial slope within a predefined range [typically 0°–3° or 5°–7°, depending on prosthesis design and posterior cruciate ligament (PCL) status], KA seeks to replicate the patient's native posterior tibial slope (PTS), which can exhibit considerable variability, reaching values as high as 20° [5, 25]. Precise restoration of the PTS is critical for appropriate PCL tensioning and for minimizing stress and wear on the posterior region of the polyethylene insert, while also ensuring stable fixation on the dense subchondral bone [19, 29]. However, excessive PTS may disrupt normal knee biomechanics, leading to increased anteroposterior instability and potentially compromising implant longevity [24, 31]. KA aims to replicate the prearthritic anatomy in every knee by also reproducing the prearthritic PTS And DFF; [20] however, in the era personalized alignment, it is still unclear whether reproducing native flexion angles for the implant positioning has an impact on clinical outcomes and implant survival. This study aimed to evaluate the impact of the variation in combined flexion (CF) and tibial posterior slope on clinical outcomes and complications in patients undergoing TKA utilizing unrestricted KA. The hypothesis was that this alignment philosophy would reveal minimal complication rates and satisfactory clinical outcomes regardless of the CF and PTS, thus demonstrating that restoring the pre‐arthritic anatomy does not represent a risk when positioning the femoral and tibial components on the sagittal plane.

## MATERIALS AND METHODS

**Figure 1:**
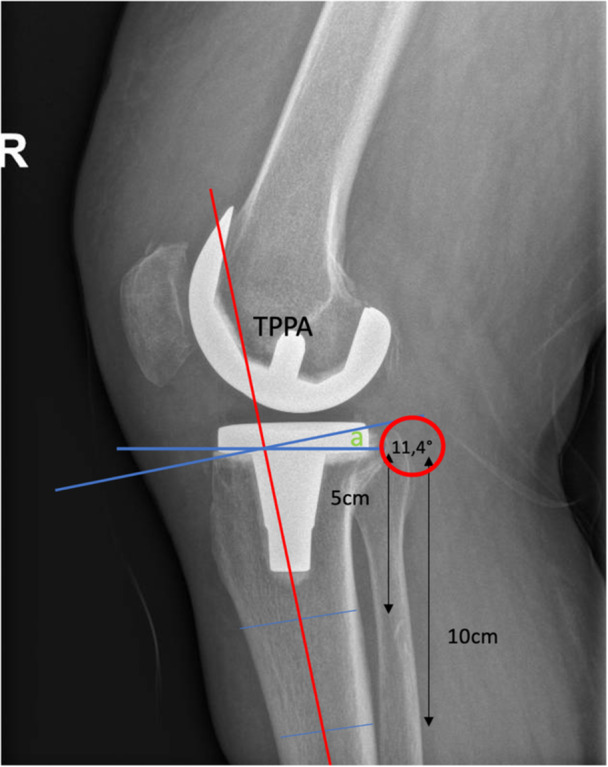
The tibial proximal anaatomic axis (TPAA) is determined by connecting the midpoints between the anterior and posterior tibial cortical at a distance of 5 and 10 cm from the joint line. Then the slope angle (a) is derived between the tangent to the tibial plateau and the line perpendicular to the TPAA.

**Figure 2:**
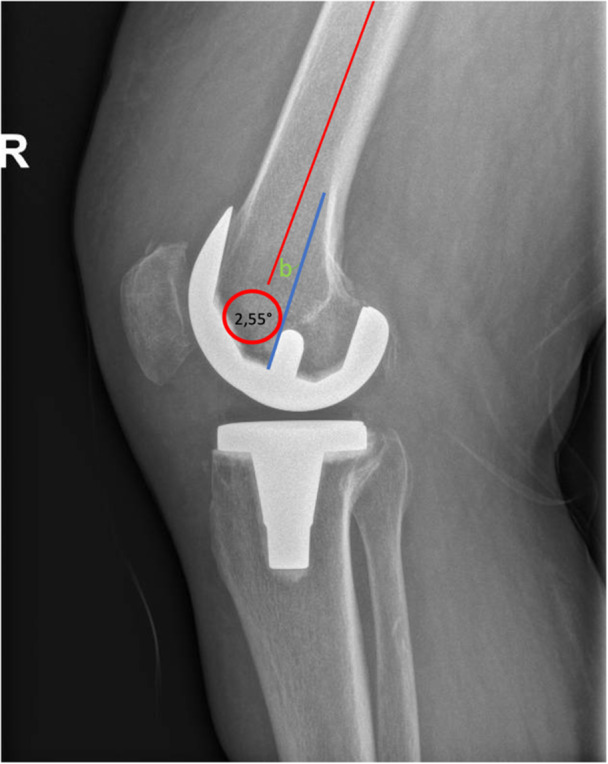
The sagittal alignment of the femoral component (b) can be determined by measuring the angle between the sagittal femoral anatomic axis (SFAA) and the femoral stem.

This study, designed retrospectively using a prospectively maintained database, the FP‐UCBM database, included all patients who underwent primary TKA conducted according to unrestricted KA performed in a high‐volume center specialized in primary arthroplasty, between November 2022 and June 2023. Patients were operated by the senior surgeons of the group (E.F., R.P., S.C.) All patients received a cruciate‐retaining Medacta GMK Sphere Total Knee System implant (Medacta USA, Inc.), using a cruciate‐retaining insert. Institutional review board approval was granted for this research (IRB No. 32.19 OSS) and all participants provided informed consent. Of the initial cohort of 172 patients, exclusions were made for those with missing preoperative, intraoperative, or follow‐up data essential to the study (24 patients), as well as those lacking complete radiographic documentation (11 patients). Demographic data, such as age, gender and body mass index (BMI), were collected. Preoperative evaluations for all patients included measurements of knee range of motion (ROM) and the Knee Society Score (KSS) pt. 1 (Objective) and pt. 2 (Functional) [33] Oxford Knee score (OKS) [28] and Short Form 12 (SF12) [8]. Imaging evaluations were conducted using anteroposterior, lateral, Rosenberg, sunrise, and full‐length weight‐bearing radiographs, performed preoperatively, at 1 month, and at 12 months of follow‐up. From these, the mechanical hip–knee–ankle angle (mHKA), lateral distal femoral angle (LDFA) and the medial proximal tibial angle (MPTA) were calculated. Immediate Postoperative imaging consisted of short anteroposterior and lateral radiographs of the knee only. From these, tibial slope (PTS), as described by Dejour et al. [9] (Figure 1) and the DFF [10] (Figure 2) were calculated. DFF resulted in flexion (positive) values/extension (negative) values based on the mechanical axis. The minimum clinical follow‐up was 1 year. At the final clinical follow‐up (12 months), clinical evaluations included knee ROM, the KSS pt. 1 (Objective) and pt. 2 (Functional), OKS and SF12 and the Forgotten Joint Score (FJS‐12) [7]. Data on complications were collected by eventual notification until August 2025 (26 months minimum follow up). The complications collected were: periprosthetic joint infection, superficial wound infection, bleeding, hematoma formation, deep vein thrombosis, pulmonary embolism, wound healing problems, neurovascular injury such as common peroneal nerve palsy or vascular damage, joint stiffness or arthrofibrosis, prosthetic loosening (either septic or aseptic), periprosthetic fractures, joint instability, patellofemoral complications such as patellar maltracking, patellar clunk syndrome, or patellar fracture and heterotopic ossification. Patients were divided into groups to analyze the impact of implant positioning in relation to the sagittal plane. With radiographic evaluation on PTS and distal DFF, the CF was calculated. CF is determined by the addition between flexion of the tibial and femoral components. Based on CF, the group with values ≤7.5° (Group A) was compared to the group with values >7.5° (Group B) [1]. For the evaluation of PTS, three groups were compared: <5 (Group C), between 5 and 10 (Group D) and >10 (Group E). These groups, within the two respective analyzed parameters, were compared in terms of clinical outcomes, radiographic measurements and complication rates. All measurements were performed by two independent investigators (P.G. and M.S.) using tools available in a picture‐archiving communication system and recorded to the nearest 0.1°.

### Principles of sagittal plane management in Unrestricted Kinematic Alignment

KA aims to replicate the prearthritic anatomy of each individual knee. Regarding the DFF, since the native DFF is often close to 0°, the technique seeks to reproduce this by minimizing flexion of the femoral component. This is achieved by slightly anteriorizing the entry point of the femoral intramedullary rod, thereby allowing guidance from the anterior femoral cortex and reducing alignment error [20].

Moreover, small deviations in femoral component flexion are unlikely to result in significant biomechanical alterations due to the ‘single radius’ design of the femoral component [38]. To restore the PTS, different techniques could be used. The conventional KA technique positions the angel wing through the cutting slot of the tibial guide, aligned along the medial border of the medial tibial plateau. The posterior slope is then determined solely by visual assessment, adjusting the plane of the angel wing to match the posterior inclination of the medial joint line. However, for the present cohort, the ‘over‐the‐top’ technique described by Franceschetti et al. [13] was used. The latter involves using an angel wing placed through the cutting slot of the tibial guide and over the anterior and posterior rims of the medial tibial plateau to set the PTS: an angel wing is inserted into the cutting slot of the tibial guide and placed ‘over‐the‐top’ of the medial tibial plateau. Then, the slope is adjusted until the angel wing touches both the anterior and posterior rims of the medial tibial plateau. This technique was shown to offer enhanced accuracy and consistency in restoring the native PTS when compared to the conventional KA method and can be reliably utilized even by less experienced surgeons. An analysis of slope reproducibility between preoperative and postoperative measurements was conducted to assess the accuracy of reproducing each patient's native slope. Variations greater than 2° from the preoperative value were classified as outliers.

### Statistical analysis

Descriptive statistics were performed to describe mean, standard deviation (SD) and range for all variables. All data analyses were performed using STATA 18 Software (StataCorp LLC). Repeated‐measures analysis of variance (ANOVA) was used to evaluate the effects of CF and PTS on KSS, OKS, SF‐12 and FJS scores. Comparisons of categorical variables between the two CF groups, including complication rates, implant revision rates, reoperation rates, and causes of mechanical failure, were performed using the chi‐square test. The significance level was set at *p* = 0.05. A post hoc power analysis for the FJS comparison between Group A (65 patients, 90.7 ± 20.8) and Group B (72 patients, 93.8 ± 16.2) indicated a statistical power of 15% (a 15% chance of finding a statistical difference with an alpha of 0.05), reflecting the very small difference between groups rather than the sample size.

## RESULTS

137 patients were evaluated with a mean follow‐up period of 1.85 ± 0.45 years. The cohort included 83 females (60.6%) and 54 males (39.4%), with an average age of 70.3 ± 7.0 years. The mean, SD and range of CF, PTS and DFF were 8.2° ± 3.0 (0.1–17.5), 5.8° ± 2.8 (0.2–14.8) and 2.6° ± 3.1 (−4.2−7.6), respectively. No surgical procedures were performed on the patella in any of the patients included in the study. At final follow‐up, three major complications were recorded, resulting in an overall complication rate of 2.2%. Specifically, one patient (0.7%) underwent revision surgery for femoral component failure, another (0.7%) required reoperation for acute infection managed with the DAIR procedure (debridement, antibiotics and implant retention), and one patient (0.7%) underwent manipulation under anesthesia. The cause of the partial revision was a medial deviation of the prosthetic trochlear angle of the femoral component relative to the quadriceps vector, which led to patellar subluxation during flexion–extension and anterior knee pain. This complication is well documented with this alignment technique [21]. Replacing the femoral component with a specific implant optimized for kinematic alignment (KA) resolved both the biomechanical issue and the patient's symptoms. The overall implant survival rate at final follow‐up was 99.3%, with only 0.7% of patients requiring partial or complete revision of the prosthesis. Inter‐rater reliability of the measurements was measured using the kappa (*κ*) statistic [22]. The resulting inter‐rater agreement *K* value was 0.92.

### CF of the implant

**Figure 3:**
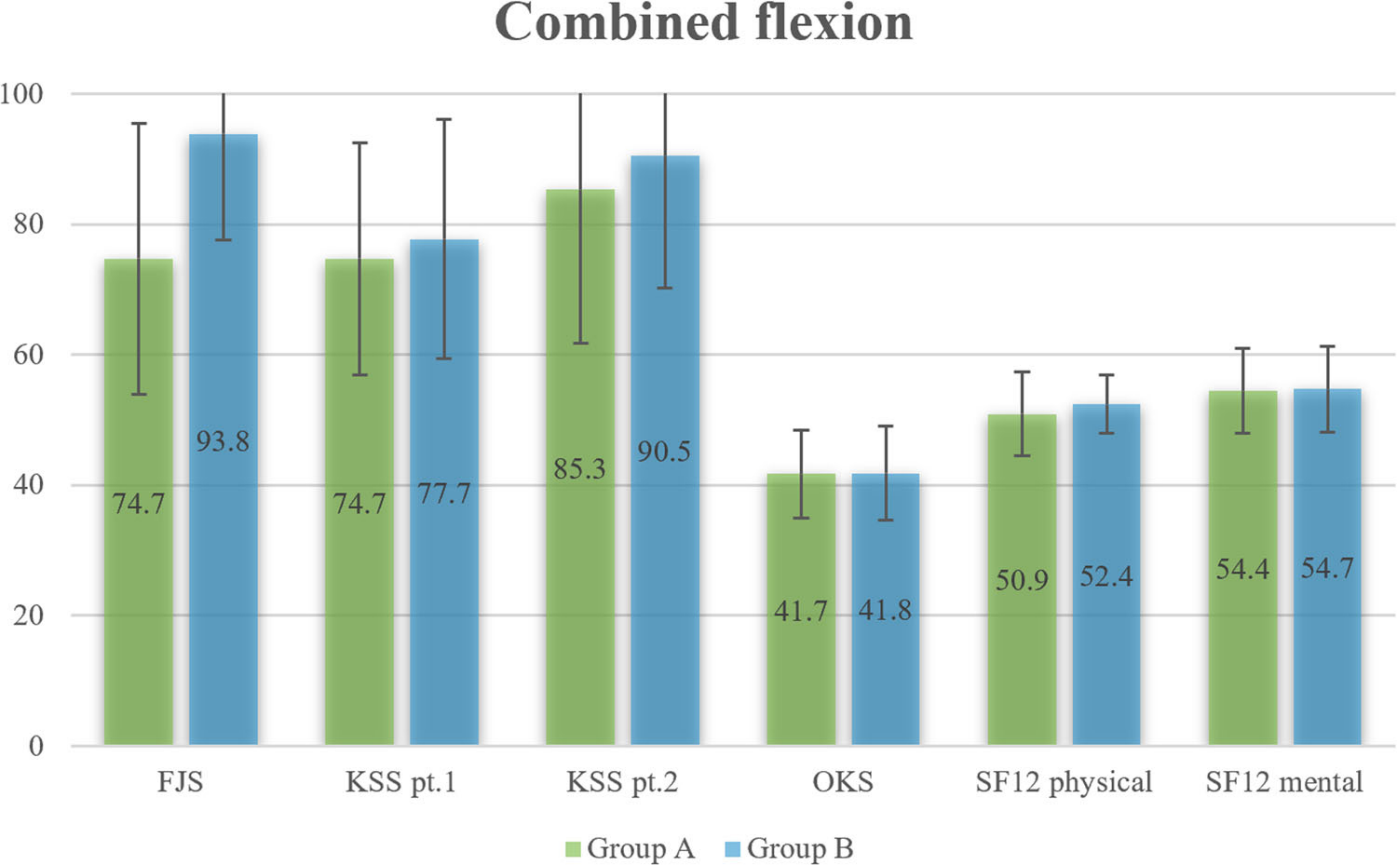
Postoperative outcomes between group A and group B.

Group A and Group B consisted of 65 (47.8%) and 72 (52.2%) patients, respectively. The mean, SD and range of CF for the two groups are reported in Table 1. Comparative analysis between the two groups showed no statistically significant differences in mean follow‐up duration, age, sex, or BMI. Similarly, preoperative radiographic parameters, including mHKA (*p* = 0.634), LDFA (*p* = 0.419), and MPTA (*p* = 0.412), were comparable (Table 1). No significant preoperative differences were found in clinical scores (overall *p* = 0.382) (Table 2). At final follow‐up, clinical outcomes remained statistically similar between groups (Table 3 and Figure 3). There were no significant differences in overall complication rates (3.2% vs. 1.4%; *p* = 0.581), implant revision rates (1.5% vs. 0%; *p* = 0.621), re‐operation rates (3.2% vs. 1.4%; *p* = 0.581), or causes of mechanical failure (1.5% vs. 0%; *p* = 0.621). Finally, no significant differences were found in the comparison of preoperative to postoperative score differences between groups (Table 4).

**Table 1:** Demographic characteristics of the CF groups.

Combined flexion	Age ± SD (years)	BMI ± SD (kg/m^2^)	Gender [female, *n* (%)]	mHKA ± SD (°)	LDFA ± SD (°)	MPTA ± SD (°)
Group A: ≤7.5°	70.4 ± 7.5	29.7 ± 4.1	38 (59.4)	−3.5 ± 6.5	88.7 ± 2.5	87.3 ± 2.6
Group B: >7.5°	70.2 ± 7.1	29.4 ± 5.3	41 (64.1)	−3.7 ± 6.2	88.5 ± 2.3	87.1 ± 2.1

Abbreviations: CF, combined flexion; LDFA, lateral distal femoral angle; MA, mechanical alignment; mHKA, mechanical hip‐knee‐angle; MPTA, medial proximal tibial angle.

**Table 2:** Mean, SD and range and preoperative clinical scores of the CF groups (mean ± SD).

Combined flexion	Mean ± SD (range)					
Group A: ≤7.5°	5.5° ± 1.6 (0.1–7.5)					
Group B: >7.5°	10.3° ± 2.1 (7.6–17.5)					

	KSS pt.1	KSS pt.2	OKS	SF12 physical	SF12 mental	Overall *p* value
Group A: ≤7.5°	38.2 ± 18.6	50.6 ± 18.1	19.8 ± 7.1	32.8 ± 9.1	45.5 ± 11.6	0.382
Group B: >7.5°	35.4 ± 19.5	48.2 ± 19.8	19.3 ± 8.6	31.8 ± 9.3	44.9 ± 14.0	

Abbreviations: CF, combined flexion; OKS, Oxford Knee Score.

**Table 3:** Postoperative clinical scores for each group (mean ± SD).

Combined flexion	KSS pt.1	*p* value	KSS pt.2	*p* value	OKS	*p* value	SF12 physical	*p* value	SF12 mental	*p* value	Forgotten Joint Score	*p* value
Group A: ≤7.5°	74.7 ± 17.8	0.427	85.3 ± 23.6	0.212	41.7 ± 6.7	0.858	50.9 ± 6.4	0.138	54.4 ± 6.5	0.982	90.7 ± 20.8	0.421
Group B: >7.5°	77.7 ± 18.3		90.5 ± 20.3		41.8 ± 7.2		52.4 ± 4.5		54.7 ± 6.6		93.8 ± 16.2	
**Tibial slope**												
<5.0° (*n* = 47)	75.1 ± 17.8	0.621	86.2 ± 23.6	0.707	41.7 ± 6.8	0.548	51.1 ± 6.5	0.952	54.4 ± 6.6	0.606	90.9 ± 20.7	0.805
5.0–10.0 (*n* = 64)	76.1 ± 18.1		87.9 ± 22.6		41.8 ± 7.2		51.3 ± 5.7		54.6 ± 6.5		91.9 ± 18.8	
>10.0° (*n* = 26)	76.5 ± 18.3		88.9 ± 21.1		41.8 ± 7.1		52.1 ± 4.8		54.7 ± 6.6		93.1 ± 16.3	

**Table 4:** Comparison of preoperative to postoperative score differences for each group (mean ± SD).

Combined flexion	Knee Society Score (KSS) pt.1	*p* value	KSS pt.2	*p* value	Oxford Knee Score	*p* value	Short Form 12 (SF12) physical	*p* value	SF12 mental	*p* value
Group A: ≤7.5°	35.6 ± 23.5	0.159	34.7 ± 27.4	0.135	21.9 ± 8.2	0.888	18.1 ± 9.9	0.159	8.9 ± 13.4	0.881
Group B: >7.5°	42.3 ± 27.2		42.3 ± 27.7		22.5 ± 10.4		20.7 ± 9.9		9.8 ± 14.0	
**Tibial slope**										
<5.0° (*n* = 47)	37.6 ± 25.8	0.639	36.1 ± 27.4	0.644	22.0 ± 9.9	0.572	18.6 ± 11.2	0.809	9.1 ± 13.4	0.586
5.0–10.0 (*n* = 64)	39.6 ± 26.2		39.0 ± 29.2		22.3 ± 10.6		19.3 ± 10.8		9.6 ± 13.6	
>10.0° (*n* = 26)	40.5 ± 26.7		40.4 ± 28.4		22.4 ± 10.7		20.3 ± 10.5		9.9 ± 15.0	

### Variation in PTS

**Figure 4:**
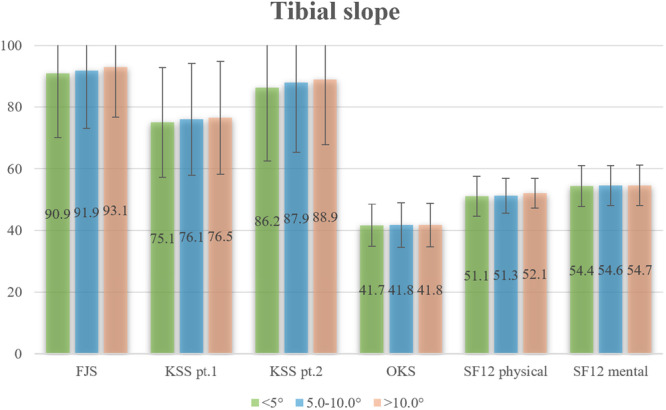
Postoperative outcomes stratified by tibial slope groups. FJS, Forgotten Joint Score; KSS, Knee Society Score; OKS, Oxford Knee Score; SF12, Short Form 12.

In the present cohort of patients, the analysis of slope reproducibility between preoperative and postoperative measurements yielded comparable results, with 0% classified as outliers. Group C (PTS <5) consisted of 47 patients (34.3%), Group D (PTS: 5–10) of 64 patients (46.7%) and Group E (PTS >10) of 26 patients (19%). The mean, SD and range of PTS for each group are reported in Table 3. In Figure 4, the distribution of the clinical scores between groups is represented, no statistically significant differences were identified among the three groups concerning, preoperative clinical scores (Table 5), clinical scores (Table 3) and preoperative to postoperative scores (Table 4). There were no statistically significant differences observed in the overall complication rates (2.1% vs. 3.1% vs. 0%; *p* = 0.683), the causes of mechanical failure (0% vs. 1.5% vs. 0%; *p* = 0.592), the re‐operation rates (2.1% vs. 3.1% vs. 0%; *p* = 0.683), or the implant revision rates (0% vs. 1.5% vs. 0.0%; *p* = 0.592).

**Table 5:** Mean, SD, range of PTS and preoperative clinical scores (mean ± SD) of the PTS groups.

Tibial slope	Mean ± SD (range)					
<5.0° (*n* = 47)	2.8 ± 1.2° (0.2–4.9)					
5.0–10.0° (*n* = 64)	7.1 ± 1.3° (5.1–10.0)					
>10.0° (*n* = 26)	12.1 ± 1.7° (10.1–14.8)					

**Tibial slope**	KSS pt.1	KSS pt.2	OKS	SF12 physical	SF12 mental	Overall *p* value
<5.0° (*n* = 47)	37.5 ± 18.6	50.1 ± 18.2	19.7 ± 7.2	32.5 ± 9.1	45.3 ± 11.7	
5.0–10.0° (*n* = 64)	36.5 ± 19.0	48.9 ± 18.5	19.5 ± 7.8	32.0 ± 9.2	45.0 ± 12.0	0.403
>10.0° (*n* = 26)	36.0 ± 19.5	48.5 ± 19.0	19.4 ± 8.0	31.8 ± 9.3	44.8 ± 13.5	

Abbreviations: KSS, Knee Society Score; PTS, posterior tibial slope; SF12, Short Form 12.

## DISCUSSION

The main finding of this study was that variations in distal femoral and tibial flexion angles when performing kinematic alignment do not influence clinical outcomes or complication rates. Comparable clinical benefits were observed irrespective of the patients' CF angles (CF ≤7.5° vs. CF >7.5°) or PTS (0°–5° vs. 5°–10° vs. >10°). This is the first study to stratify the clinical impact of CF or the variation of PTS on patients undergoing the unrestricted kinematic alignment technique. Whereas other patient‐specific techniques rely on fixed boundaries of final sagittal alignment to guide implant positioning [35, 36], KA aims to replicate the prearthritic sagittal anatomy of each knee [20]. However, given that native DFF is typically near 0°, the technique aims to replicate this anatomical orientation by minimizing flexion of the femoral component. In contrast, the technique allows unrestricted replication of the PTS, which can demonstrate substantial variability, with values reaching up to 20° [5, 25].

### Role of sagittal femoral alignment

The role of sagittal femoral alignment in enhancing postoperative function is well described. Nishitani et al. found that patients with slightly flexed femoral implants had better functional outcomes one year after TKA than those with extended or excessively flexed implants [30]. Likewise, Hassan et al. observed that, after a 2‐year follow‐up, femoral component flexion between 0° and 3° was linked to patients reporting their knee felt consistently ‘normal’ [11]. Wang et al. investigated 120 knees treated with robotic‐assisted TKA, categorizing them into two groups: one receiving individualized femoral flexion and the other using a standard flexion angle of 3°–5°. The results showed that the individualized group had a significantly lower incidence of femoral component extension and a higher proportion of cases achieving optimal prosthesis flexion between 0° and 3° [39]. Murphy et al. [27] performed a double‐blind, randomized controlled trial involving cruciate‐retaining TKA patients, comparing femoral component positioning at 0° versus 4°. Outcome measures included knee flexion and extension, WOMAC, SF‐12, the timed stand test, stair climb test, and overall patient satisfaction at one year postoperatively. The group with the femoral component positioned at 4° demonstrated significantly greater knee flexion and higher SF‐12 scores, while no other measures showed significant differences. Antony et al. [3] identified a weak positive correlation between femoral component flexion and both maximum knee flexion and overall ROM. However, elevated failure rates have been reported in cases where the femoral component was flexed beyond 3°. Although increased flexion may enhance postoperative knee mobility, the associated risk of early aseptic failure appears to outweigh its potential functional benefits. Then, the ideal sagittal positioning of the femoral component in TKA continues to be a subject of investigation, especially in evaluating the effectiveness of standardized alignment compared to an individualized strategy [6].

### Role of sagittal tibial alignment

The majority of TKA implant manufacturers provide specific guidelines for tibial resection slope tailored to their systems. However, these standardized recommendations may not correspond with the patient's native PTS, which exhibits considerable interindividual variability [15]. Cadaveric studies have demonstrated that each 1° increase in PTS results in an average increase of approximately 1.7° in knee flexion. This effect is attributed to delayed impingement between the tibial insert and the femoral component [4]. Nonetheless, clinical studies examining the relationship between PTS and knee function have produced inconsistent findings. Sinno et al. assessed 168 PS TKA patients using the SF‐12 mental and physical component scores alongside the functional KSS. Their analysis revealed that when PTS exceeded 5°, functional outcomes were comparable between PS and CR implants. However, patients with PS TKA and PTS less than 5° demonstrated inferior functional results [37]. Seo et al. [34], in a study of 801 CR TKA cases, found no significant differences in KSS knee and functional scores or ROM among various PTS groups. However, patients with a ΔPTS between −1° and 3° demonstrated superior Feller patella scores and Kujala scores. Lee et al. analyzed 164 knees and found no significant changes in ROM, KSS or FJS associated with tibial slope variations. However, multivariate analysis identified a weak positive correlation between TS changes and patient‐reported difficulties in rising from sitting (WOMAC) as well as joint awareness when climbing stairs (FJS) [24]. Nedopil et al. [29] also proposed that excessive tibial slope could elevate the risk of tibial component loosening, primarily due to failure from anteroposterior tilt. However, this risk appears to be influenced by the specific implant design. Fujito et al., in a study of 71 CR TKA cases featuring high geometric conformity to the medial articular surface, observed no significant differences in complication rates, even among patients with PTS up to 10° [14]. Similarly, Richardson et al., using radiostereometric analysis of 200 TKA cases, reported no correlation between postoperative PTS and overall implant migration, anteroposterior tilt migration, or inducible displacement. Other studies have indicated that an increased PTS reduces joint contact stress, decreases quadriceps muscle force demands during knee extension, and minimizes posterior femoral impingement [23, 26]. Although alterations in PTS can affect knee kinematics and stability, the clinical significance remains unclear. While excessive PTS may result in abnormal anteroposterior translation and paradoxical motion, moderate adjustments in PTS have the potential to enhance stability and improve quadriceps efficiency following TKA. However, most of the reported studies refer to a standardized technique (MA), therefore the interpretation of the results should be approached with caution.

### Role of CF

To our knowledge, the analysis by Andriollo et al. [1] represents the sole study assessing the impact of CF of the femoral and tibial components in TKA using a personalized alignment strategy (robotic‐assisted functional alignment). Their work also evaluated deviations from native anatomy and examined correlations with functional outcomes and complication rates. Consistent with our findings, they reported no significant differences in functional scores, ROM, or complication rates among 310 patients, irrespective of flexion values. These results support the hypothesis that a personalized sagittal alignment approach can be safely incorporated into emerging TKA alignment techniques.

### Limitations

The present study has several limitations. First, the short follow‐up period should be acknowledged when considering revision or complication rates. A major concern with new alignment philosophies is the risk of wear and/or loosening of the prosthesis, which requires long‐term follow‐up for proper evaluation. Also, registry follow up as a single‐center retrospective investigation, it lacks the methodological rigor and generalizability associated with prospective, multicenter studies. However, this design allows for homogeneous data collection and evaluation of a cohort of osteoarthritic patients treated with a consistent surgical technique by a limited number of experienced surgeons. Such an approach enhances the immediate interpretability of the results by minimizing confounding external factors. Additionally, the use of a single implant model may limit the reproducibility of these findings in patients with different prosthetic configurations. Further studies are needed to evaluate potential clinical variations, mechanical or radiological complications in the medium‐to‐long term follow up in such cohort of patients.

## CONCLUSION

The study highlights that personalized sagittal alignment, following the principles of unrestricted KA, allows for comparable results in terms of complication rates, overall clinical outcomes, and patient‐reported satisfaction. Furthermore, this technique seems to offer satisfactory outcomes in the short‐term regardless the sagittal orientation of the components. Therefore, restoring the prearthritic sagittal orientation of the components is considered safe and does not require adherence to predefined ‘safe zones’ when using unrestricted kinematic alignment in the short term. A longer follow‐up period is required to validate these findings over the medium and long term.

## AUTHOR CONTRIBUTIONS

Pietro Gregori and Edoardo Franceschetti had the idea for the article and were responsible for revising the manuscript. Pietro Gregori was responsible for writing of the manuscript and qualified as corresponding author. Marco Spatuzzi and Andrea Tanzilli were responsible for data acquisition and analysis and realisation of Figures and Tables. Andrea Tanzilli was responsible of statistical analysis. Biagio Zampogna, Umile Giuseppe Longo and Rocco Papalia were responsible for conceptualisation and supervised data acquisition and analysis. Giancarlo Giurazza, Stefano Campi and Matteo Formica were responsible for reviewing and critically revise the manuscript. All authors have given final approval of the version to be published.

## CONFLICT OF INTEREST STATEMENT

The authors declare no conflicts of interest.

## ETHICS STATEMENT

All procedures adhered to the ethical standards set by the institutional and/or national research committee, as well as the 1964 Helsinki Declaration and its subsequent amendments or equivalent ethical guidelines. Institutional review board approval was granted for this research (IRB No. 32.19 OSS) and all participants provided informed consent. All patients provided legitimate informed consent.

## Data Availability

The data that support the findings of this study are available from the corresponding author [P. G.], upon reasonable request.
